# Association between metabolic dysfunction-associated steatotic liver disease and insulin resistance in individuals with normoglycemia

**DOI:** 10.1097/MD.0000000000044109

**Published:** 2025-08-22

**Authors:** Nai-Hui Liao, Chun-Yi Wang, Kuan-Yu Lai, Hsiang-Han Kao, Wen-Yuan Lin, Tsung-Po Chen

**Affiliations:** a Department of Family Medicine, China Medical University Hospital, Taichung, Taiwan; b School of Medicine, College of Medicine, China Medical University, Taichung, Taiwan.

**Keywords:** insulin resistance, metabolic dysfunction-associated steatotic liver disease, obesity, type 2 diabetes

## Abstract

Metabolic dysfunction-associated steatotic liver disease (MASLD) is increasingly prevalent, especially among individuals with type 2 diabetes mellitus. Insulin resistance (IR) is a key shared mechanism. This study aimed to investigate the association between MASLD and IR in individuals with normal glucose levels. We recruited participants from communities and outpatient clinics in central and northern Taiwan, excluding those with impaired fasting glucose or type 2 diabetes mellitus. Data collected included body mass index, blood pressure, body fat percentage, and lifestyle factors. After overnight fasting, blood samples were taken. MASLD was diagnosed via abdominal ultrasonography. IR was calculated using the homeostasis model assessment of insulin resistance formula and categorized into tertiles (T1–T3). Statistical analyses included analysis of variance and multivariate logistic regression. Among 485 participants (mean age 42.0 ± 11.4 years), MASLD prevalence increased with IR levels (T1: 34.1%, T2: 40.1%, T3: 47.8%; *P* < .001). Fasting glucose remained within normal limits but rose with IR (T1: 81.9 ± 7.4, T2: 85.1 ± 7.6, T3: 87.3 ± 6.5 mg/dL; *P* < .0001). After adjusting for age, sex, body mass index, exercise, alcohol use, and smoking, MASLD risk was significantly higher in T3 (odds ratio: 3.87, 95% confidence interval: 2.08–7.21) and T2 (odds ratio: 1.83, 95% confidence interval: 1.11–3.00) compared to T1. Even among individuals with normal glucose levels, elevated IR is significantly associated with higher MASLD risk. Early identification and intervention for IR may help prevent MASLD progression.

## 1. Introduction

Metabolic dysfunction-associated steatotic liver disease (MASLD), previously known as nonalcoholic fatty liver disease, has recently been redefined to better reflect the metabolic underpinnings of liver fat accumulation.^[1]^ This change in nomenclature, endorsed by leading international hepatology societies, acknowledges the central role of metabolic dysfunction in the pathogenesis of hepatic steatosis. MASLD now encompasses individuals with hepatic fat accumulation and at least 1 cardiometabolic risk factor, including insulin resistance, obesity, dyslipidemia, or hypertension, regardless of alcohol intake.^[2]^

The global prevalence of MASLD among individuals with type 2 diabetes has been reported to range between 60% and 86%.^[3]^ Patients with MASLD have a 2-fold higher risk of developing type 2 diabetes compared to those without MASLD, and more severe liver disease may further increase this risk.^[4]^ Insulin resistance (IR) is widely recognized as a key driver of MASLD development and progression.^[5]^ In people with overt metabolic diseases such as type 2 diabetes or obesity, IR contributes to hepatic fat accumulation through increased lipolysis, free fatty acid delivery to the liver, and de novo lipogenesis.^[6]^ These pathophysiologic mechanisms set the stage for hepatic steatosis, inflammation, and eventually fibrosis. However, the majority of studies investigating this association have primarily focused on populations with overt glucose dysregulation, including impaired fasting glucose, prediabetes, or established diabetes.^[7]^

Emerging evidence suggests that metabolic disturbances, including hepatic steatosis, may begin well before glucose dysregulation is apparent.^[8]^ Yet, few studies have specifically evaluated the relationship between MASLD and insulin resistance in individuals with normal glucose levels. This subgroup represents a clinically important but often overlooked population who may harbor early metabolic dysfunction without meeting diagnostic thresholds for hyperglycemia. If MASLD is associated with insulin resistance even in normoglycemic individuals, it may serve as a valuable early marker of metabolic disease risk—potentially preceding diabetes, cardiovascular disease, or other manifestations of metabolic syndrome.

We hypothesized that even among individuals with normal fasting plasma glucose and independent of type 2 diabetes, higher levels of IR would be associated with an increased likelihood of developing MASLD. Therefore, the aim of this study was to investigate the association between MASLD and IR in a normoglycemic adult population.

## 2. Methods

### 2.1. Study design and participants

This community-based cross-sectional study was conducted in 3 urban regions of Taiwan—Taipei City, Taichung City, and Hsinchu City—between 2014 and 2021. The study protocol adhered to the ethical guidelines of the Declaration of Helsinki and was approved by the Institutional Review Boards of National Taiwan University Hospital (IRB nos. 201210012RIC, 201705073RINC) and China Medical University Hospital (IRB no. CMUH110-REC2-064). Written informed consent was obtained from all participants prior to data collection.

### 2.2. Data collection

Data on sociodemographic characteristics, medical history, and lifestyle factors were collected using a standardized, interviewer-administered questionnaire. Information included the presence of hypertension, type 2 diabetes, dyslipidemia, and thyroid disorders. Lifestyle behaviors such as alcohol consumption, cigarette smoking, and physical activity were also recorded. Smoking status was categorized as nonsmoker, current smoker, or former smoker. Physical activity was dichotomized as ≥ 150 or < 150 min/wk.

All participants underwent standardized physical examinations following an overnight fast of at least 8 hours. Anthropometric and metabolic parameters were measured using calibrated instruments by trained personnel. Details of the data collection methodology have been published in previous studies. Those with medical history of type 2 diabetes, elevated fasting plasma glucose (≥100 mg/dL) or treatment with glucose-lowering medications were excluded from our study.

### 2.3. Assessment of insulin resistance

IR was assessed using the homeostasis model assessment of insulin resistance (HOMA-IR),^[9]^ a widely accepted surrogate marker that reflects hepatic insulin sensitivity. After an overnight fast of at least 8 hours, fasting plasma glucose (mg/dL) and fasting insulin (μU/mL) levels were measured. HOMA-IR was calculated using the following formula:


$$\begin{array}{l} HOMA\text{-}IR\;=\;fastinginsulin\;(\mu U/mL)\times fastingglucose\;(mg/dL)/405 \end{array}$$

This index has been validated in both clinical and epidemiological studies and is considered a practical and reliable method for estimating insulin resistance, particularly in large population-based research settings.

To analyze the association between insulin resistance and MASLD, participants were stratified into tertiles based on HOMA-IR values. Tertile 1 (T1) represented the lowest third of HOMA-IR values, tertile 2 (T2) the middle third, and tertile 3 (T3) the highest third. This stratification allowed us to evaluate dose–response relationships between increasing levels of insulin resistance and the risk of MASLD.

### 2.4. Assessment of hepatic steatosis

Abdominal ultrasonography was performed after an 8-hour fast using a high-resolution B-mode scanner (Hitachi Aloka ProSound α6 or GE Voluson E8) equipped with a 3.5 to 5 MHz transducer. Hepatic steatosis was assessed using the ultrasound fatty liver index (US-FLI),^[10]^ a validated scoring system ranging from 0 to 8. Detailed methods were reported in our previous publication.^[11,12]^ A US-FLI score ≥ 2 was used to diagnose hepatic steatosis, which corresponds to a histologic steatosis ≥ 10%, with a sensitivity of 90.1% and specificity of 90%.

All ultrasound examinations were performed by 3 experienced physicians across the 3 cities, who received unified training in image acquisition and US-FLI scoring to ensure inter-observer consistency.

### 2.5. Diagnosis of MASLD

MASLD was diagnosed based on the presence of hepatic steatosis in combination with at least 1 cardiometabolic risk factor, following the 2023 international consensus criteria. Hepatic steatosis was identified using abdominal ultrasonography, with a US-FLI score of 2 or higher indicating the presence of steatosis. Given that all participants were normoglycemic by study design (fasting plasma glucose < 100 mg/dL), the fasting glucose criterion for MASLD was not applicable. Thus, MASLD was defined as the presence of hepatic steatosis (US-FLI ≥ 2) plus at least one of the following: overweight/obesity (body mass index [BMI] ≥ 24 kg/m^2^), elevated blood pressure (systolic ≥ 130 mm Hg, diastolic ≥ 85 mm Hg, or antihypertensive use), or abnormal lipid profile (triglycerides ≥ 150 mg/dL or high-density lipoprotein cholesterol < 40 mg/dL in men and < 50 mg/dL in women). Individuals with significant alcohol intake, viral hepatitis, or other known causes of secondary hepatic steatosis were excluded to ensure that the diagnosis reflected primary metabolic dysfunction-associated liver disease.

### 2.6. Statistical analysis

Categorical variables were summarized as frequencies and percentages, while continuous variables were expressed as mean ± standard error. Group comparisons were performed using analysis of variance for continuous variables and the chi-square test for categorical variables.

Multivariate logistic regression models were used to evaluate the association between IR and MASLD, adjusting for potential confounders, including age, sex, smoking status, alcohol consumption, and physical activity. Odds ratios (ORs) and 95% confidence intervals (CIs) were calculated. A 2-tailed *P*-value < .05 was considered statistically significant. All statistical analyses were conducted using SAS software, version 9.4 (SAS Institute Inc., Cary, NC).

## 3. Result

**Figure 1.**
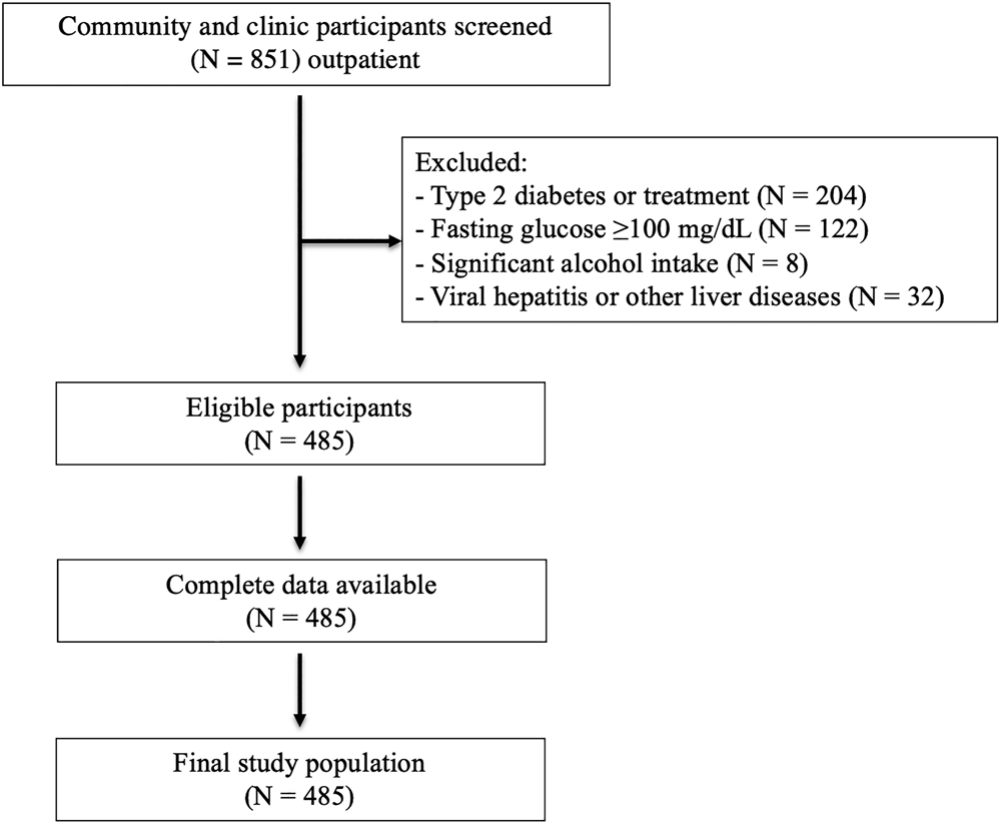
Participant flow diagram.

A total of 851 participants were screened, of whom 366 were excluded based on predefined criteria (Fig. 1). Of the 485 participants who met the inclusion criteria, all had complete data for the variables used in this analysis. Therefore, no imputation was required. A total of 485 normoglycemic participants were included in the analysis and stratified into tertiles based on HOMA-IR values to assess the relationship between IR and MASLD. Table 1 summarizes the demographic, anthropometric, metabolic, and lifestyle characteristics across tertiles.

**Table 1:** Baseline clinical and biochemical characteristics of the study subjects.

	Tertile 1	Tertile 2	Tertile 3	*P*-value	
	n = 167	n = 157	n = 161		
Age (yr)	41.6 ± 11.2	42.8 ± 11.7	41.6 ± 11.0	.55	I ≠ III, I ≠ II, II ≠ III
Sex (men)	34.1%	40.1%	47.8%	.04	
Weight (kg)	58.6 ± 11.6	63.0 ± 11.0	74.9 ± 15.5	<.0001	
Height (cm)	162.7 ± 8.8	163.3 ± 8.6	165.3 ± 8.8	.02	I ≠ III
Waist (cm)	76.3 ± 7.6	80.9 ± 8.3	90.3 ± 10.5	<.0001	
Body mass index (kg/m^2^)	22.1 ± 3.5	23.5 ± 3.1	27.2 ± 4.2	<.0001	
Fat (%)	25.8 ± 7.2	28.2 ± 6.6	32.5 ± 7.5	<.0001	
Smoking	13.8%	14.7%	16.2%	.83	
Alcohol	21.0%	19.8%	17.4%	.71	
Systolic BP (mm Hg)	119.3 ± 16.4	122.4 ± 14.7	127.7 ± 17.2	<.0001	I ≠ III, II ≠ III
Diastolic BP (mm Hg)	74.8 ± 11.1	77.5 ± 10.1	80.9 ± 12.9	<.0001	I ≠ III, II ≠ III
Fasting plasm glucose (mg/dL)	81.9 ± 7.4	85.1 ± 7.6	87.3 ± 6.5	<.0001	
Total-C (mg/dL)	194.5 ± 36.4	196.8 ± 34.6	197.8 ± 36.3	.68	
HDL-C (mg/dL)	62.6 ± 14.1	59.2 ± 14.3	49.8 ± 11.5	<.0001	I ≠ III, II ≠ III
LDL-C (mg/dL)	121.3 ± 34.3	125.2 ± 33.0	128.6 ± 33.8	.15	
Triglyceride (mg/dL)	88.8 ± 49.6	101.8 ± 57.6	149.2 ± 110.6	<.0001	I ≠ III, II ≠ III
ALT (IU/L)	18.7 ± 11.0	23.4 ± 15.6	36.4 ± 28.5	<.0001	I ≠ III, II ≠ III
AST (IU/L)	20.2 ± 5.6	22.3 ± 8.4	25.8 ± 10.8	<.0001	I ≠ III, II ≠ III
Insulin	3.4 ± 0.8	6.2 ± 0.9	14.1 ± 7.7	<.0001	
MASLD (%)	33.5%	55.4%	83.9%	<.0001	

ALT = alanine transaminase, AST = aspartate transaminase, BP = blood pressure, HDL-C = high-density lipoprotein cholesterol, LDL-C = low-density lipoprotein cholesterol, MASLD = metabolic dysfunction-associated steatotic liver disease, Total-C = total cholesterol.

There was no significant difference in mean age across tertiles (*P* = .55), indicating that age was not a key driver of insulin resistance variation in this population. However, the proportion of male participants increased progressively across tertiles, from 34.1% in the lowest to 47.8% in the highest, with a significant difference (*P* = .04), suggesting a potential sex-related pattern in IR distribution.

Anthropometric indices showed a clear increasing trend with IR resistance. Body weight increased from 58.6 ± 11.6 kg in tertile 1 to 74.9 ± 15.5 kg in tertile 3 (*P* < .0001), while waist circumference rose from 76.3 ± 7.6 to 90.3 ± 10.5 cm (*P* < .0001). BMI and body fat percentage similarly increased across tertiles (*P* < .0001 for both), highlighting a strong association between adiposity and insulin resistance. Height showed a modest but statistically significant increase (*P* = .02).

Fasting plasma glucose levels were significantly different across tertiles, despite all participants having values within the normoglycemic range. The mean glucose level increased from 81.9 ± 7.4 mg/dL in tertile 1 to 87.3 ± 6.5 mg/dL in tertile 3 (*P* < .0001), suggesting that even within normal limits, insulin resistance is associated with a subtle upward shift in fasting glucose.

No significant differences were observed in smoking (*P* = .83) or alcohol consumption (*P* = .71) across tertiles, indicating that these lifestyle factors were unlikely to confound the observed metabolic trends.

These findings underscore a strong correlation between increasing insulin resistance and key metabolic and anthropometric abnormalities—even among individuals with normal glucose levels—supporting the relevance of early metabolic screening for MASLD risk.

The results of the logistic regression analysis examining the association between insulin resistance tertiles and the presence of MASLD are presented in Table 2. In unadjusted analysis (model 1), participants in the second HOMA-IR tertile had 2.46 times higher odds of having MASLD compared to those in the first tertile (OR = 2.46, 95% CI: 1.57–3.86). The risk increased markedly in the third tertile, with an odds ratio of 10.29 (95% CI: 6.07–17.46), indicating a strong dose–response relationship between insulin resistance and MASLD risk.

**Table 2:** multivariate logistic regression model to identify the risk of factors for MASLD.

	Model 1	Model 2	Model 3
HOMA-IR			
T2 vs T1	2.46 (1.57–3.86)	1.82 (1.11–2.98)	1.83 (1.11–3.00)
T3 vs T1	10.29 (6.07–17.46)	4.02 (2.18–7.42)	3.87 (2.08–7.21)
Sex (ref = women)		1.75 (1.10–7.42)	1.59 (0.95–2.66)
Age		1.01 (0.99–1.03)	1.01 (0.99–1.03)
Body mass index		1.29 (1.19–1.40)	1.30 (1.20–1.41)
Exercise (ref = no)			0.80 (0.51–1.25)
Smoking (ref = no)			2.02 (0.95–4.27)
Drinking (ref = no)			0.75 (0.40–1.39)

HOMA-IR = homeostatic model assessment for insulin resistance, MASLD = metabolic dysfunction-associated steatotic liver disease.

After adjusting for potential confounders including age, sex and BMI (model 2), the association remained statistically significant. The adjusted odds ratio for MASLD was 1.82 (95% CI: 1.11–2.98) for tertile 2, and 4.02 (95% CI: 2.18–7.42) for tertile 3, compared to the reference group (tertile 1).

Further adjustment for smoking status, alcohol consumption, and physical activity in model 3 did not meaningfully alter the association. The odds of MASLD remained significantly elevated in the second (OR = 1.83, 95% CI: 1.11–3.00) and third tertiles (OR = 3.87, 95% CI: 2.08–7.21), reinforcing the robustness of the relationship.

These findings indicate a clear and independent association between increasing IR and the risk of MASLD, even within a normoglycemic population. The graded increase in risk across tertiles supports the hypothesis that insulin resistance plays a key role in MASLD pathogenesis, independent of glycemic status.

## 4. Discussion

This study demonstrates a significant association between IR and MASLD in normoglycemic individuals, indicating that metabolic dysfunction can occur even in the absence of overt glucose dysregulation. Our findings demonstrate a clear dose–response association, with the prevalence of MASLD rising from 34.1% in the lowest IR tertile to 47.8% in the highest (*P* < .001), and an adjusted odds ratio of 3.87 (95% CI: 2.08–7.21) for MASLD in the highest tertile compared to the lowest after controlling for confounders such as age, sex, BMI, smoking, alcohol consumption, and physical activity. These results suggest that the critical role of IR as an early driver of hepatic steatosis, even within normal glucose ranges. MASLD may serve as an early indicator of metabolic risk, preceding the development of type 2 diabetes and cardiovascular disease.

Our results extend the literature by focusing on a normoglycemic cohort, a population less studied compared to those with established metabolic diseases. Previous research has predominantly explored MASLD in the context of type 2 diabetes (T2DM) or obesity, where the prevalence is notably high (60%–86%) and the bidirectional relationship with IR is well-documented.^[5,6,13,14]^ For instance, Mantovani et al reported that MASLD was associated with a 2.22-fold increased risk of developing type 2 diabetes, with risk escalating with disease severity.^[4]^ Barb et al reported that obesity was the primary driver of hepatic steatosis, but T2DM significantly increased the risk of fibrosis, especially in those with obesity.^[13]^ However, our study shifts the lens to an earlier stage, revealing that IR-driven hepatic steatosis is prevalent (34.1%–47.8%) even among individuals with normal glucose levels. This finding challenges the traditional view that MASLD arises mainly as a consequence of overt metabolic syndrome and supports the hypothesis that it may serve as an early marker of metabolic deterioration. The significant odds ratios across IR tertiles (T2 vs T1: OR 1.83; T3 vs T1: OR 3.87), even after full adjustment, reinforce the strength of this association. Notably, lifestyle factors such as smoking and physical activity did not confound the relationship in our analysis.

The high prevalence of MASLD among normoglycemic participants suggests that screening for hepatic steatosis and IR may help identify individuals at risk before the onset of glucose dysregulation. Current guidelines typically emphasize glucose-based markers, such as fasting glucose and HbA1c, for assessing metabolic risk.^[15]^ However, our findings support the inclusion of IR measures (e.g., HOMA-IR) and imaging-based liver assessments, such as ultrasonography, in routine health evaluations. Integrating insulin resistance measures and liver imaging into standard health assessments may provide a more comprehensive understanding of metabolic risk, facilitating earlier interventions to prevent the progression to conditions like type 2 diabetes and cardiovascular disease.^[15]^ Early detection of MASLD in normoglycemic individuals enables preemptive metabolic intervention strategies. Targeted lifestyle modifications can potentially attenuate hepatic insulin resistance, mitigating pathophysiological progression to type 2 diabetes mellitus and advanced hepatic steatosis.^[16,17]^ This aligns with the redefined MASLD nomenclature, which emphasizes metabolic dysfunction over alcohol exclusion, broadening the scope for early intervention.^[1,18]^

The dose–response relationship observed across IR tertiles also highlights a potential gradient of risk that could inform risk stratification. Individuals in the highest IR tertile (T3) exhibited a nearly 4-fold increased risk of MASLD compared to the lowest tertile (T1), suggesting that even modest elevations in IR within the normal glucose range warrant attention. This pattern is similar to the gradual metabolic changes in prediabetes, where increasing insulin resistance leads to health complications over time. Moreover, the marked progression in anthropometric measures—particularly the expansion of waist circumference from 76.3 to 90.3 cm across tertiles—demonstrates the fundamental link between visceral fat accumulation and IR in MASLD. Central obesity, a key MASLD criterion, amplifies IR by releasing pro-inflammatory cytokines and free fatty acids, further exacerbating hepatic lipid accumulation. Our findings thus reinforce the importance of targeting visceral fat reduction as a cornerstone of early MASLD management.^[19]^

The observed association aligns with the pathophysiological mechanisms linking IR to MASLD.^[5]^ Insulin resistance disrupts normal lipid metabolism by enhancing peripheral lipolysis, increasing free fatty acid flux to the liver, and promoting de novo lipogenesis.^[20–22]^ These processes lead to hepatic fat accumulation, a hallmark of MASLD, which can initiate a cascade of inflammation and fibrosis if unchecked.^[23]^ Our finding that fasting plasma glucose levels, though within normal limits, increased subtly across IR tertiles (81.9 ± 7.4 to 87.3 ± 6.5 mg/dL, *P* < .0001) supports the notion that IR exerts metabolic effects prior to crossing diagnostic thresholds for hyperglycemia. A modest glucose elevation, coupled with escalating anthropometric markers like BMI, waist circumference, and body fat percentage, suggests an early stage of metabolic disruption. These findings precede clinical prediabetes or diabetes diagnosis and align with recent research evidence.

Despite the strengths of our study, several limitations must be acknowledged. First, its cross-sectional design precludes causal inference. While we observed a strong association between IR and MASLD, longitudinal studies are needed to determine whether IR precedes and drives hepatic steatosis or if early MASLD exacerbates IR in a bidirectional manner, as suggested in T2DM. Second, our reliance on ultrasonography and the US-FLI score, while validated and practical, lacks the precision of gold-standard methods like magnetic resonance spectroscopy or liver biopsy for quantifying steatosis or detecting early fibrosis. However, ultrasonography’s high sensitivity (90.1%) and specificity (90%) for detecting ≥ 10% steatosis make it a reliable screening tool in community settings.^[10,24]^ Third, our sample was drawn from urban Taiwan, potentially limiting generalizability to rural populations or other ethnic groups within the Asia-Oceania region, where dietary habits, physical activity levels, and genetic predispositions may differ. Additionally, our dataset did not include information on dietary patterns, family history of metabolic or liver disease, or socioeconomic status. These unmeasured factors may represent potential sources of residual confounding, and future studies incorporating these variables are warranted. Finally, although we adjusted for multiple confounders, residual confounding from unmeasured factors cannot be excluded.

Our findings also raise questions about the natural history of MASLD in normoglycemic individuals. The higher glucose levels within the normal range in the upper IR tertile (87.3 vs 81.9 mg/dL) suggest a preclinical phase of metabolic stress that may eventually breach normoglycemic boundaries. Additionally, validating these findings in larger, multi-ethnic cohorts could refine MASLD diagnostic criteria for normoglycemic individuals, potentially lowering the threshold for cardiometabolic risk factors in early screening protocols.

## 5. Conclusion

This study demonstrates a significant, independent association between IR and MASLD in a normoglycemic population, with a clear dose–response effect across IR tertiles. These findings highlight MASLD as an early marker of metabolic dysfunction, even in the absence of glucose abnormalities, and advocate for proactive screening and intervention in individuals with elevated IR. By identifying at-risk individuals before overt disease onset, healthcare providers can implement preventive strategies to halt the progression of metabolic and hepatic complications. Future longitudinal studies are warranted to confirm causality, assess long-term outcomes, and optimize early management approaches for this underrecognized population.

## Author contributions

**Conceptualization:** Wen-Yuan Lin, Tsung-Po Chen.

**Methodology:** Kuan-Yu Lai, Tsung-Po Chen.

**Supervision:** Hsiang-Han Kao, Wen-Yuan Lin, Tsung-Po Chen.

**Writing – original draft:** Nai-Hui Liao, Chun-Yi Wang.

**Writing – review & editing:** Kuan-Yu Lai, Hsiang-Han Kao, Tsung-Po Chen.
